# Supplementary material for: Content-rich biological network constructed by mining PubMed abstracts
Source: BMC Bioinformatics. 2004 Oct 8;5:147. doi: 10.1186/1471-2105-5-147 (PMC528731; doi:10.1186/1471-2105-5-147)
Supplement: Additional File 2 — The original results of the above study (non-essential files are deleted to keep the file size under the limit set by BMC bioinformatics). [file 1471-2105-5-147-S2.bz2 › chilibotAdditionalFile2/dip20/305ID10428074E1302/html/APP_APOE.html]

 


 **APP** and **APOE** 
  
Found 392 abstracts in PubMed, retrieved 20.  
 

 What does Google say? 
 PDF only 
| .edu only 

---

**Interactive relationship** (e.g. stimulation, inhibition, etc)

**Neutral relationship**- The incidence was significantly higher in atherosclerotic  **apoE**  deficient  **apoE**  mice, in which 8 100% of 8 mice developed AAA  [ **APP** ] .  Ref: 12600880 Circ Res, 2003
- Phosphatidylcholine phospholipase D activity, cell membrane cholesterol, and apolipoprotein E  [ **APOE** ]  levels were associated with effects of fresh amyloid beta  [ **APP** ]  peptide 1 42on cholesterol distribution but not with effects of aged amyloid beta  [ **APP** ]  peptide 1 42arguing against a common mechanism.  Ref: 12584199 J Biol Chem< MedlineTA>J Biol Chem, 2003
- Mutations in  **APP**  and the presenilin genes account for less than 1% of the prevalence of the disease in the general population compared to 10 17% for the  **APOE**  variation.  Ref: 12629906 Tijdschr Gerontol Geriatr, 2003
- Possession of the  **APOE**  varepsilon 4 allele has also been shown to influence neuropathological findings in patients who die from traumatic brain injury, including the accumulation of amyloid beta  [ **APP** ]  protein.  Ref: 12782758 Mol Pathol< MedlineTA>Mol Pathol, 2003

**Non-interactive relationship** (e.g. studied together, co-existance, homology, etc.)

- Apolipoprotein E  [ **APOE** ]  enhances uptake of soluble but not aggregated amyloid beta  [ **APP** ]  protein into synaptic terminals.  Ref: 12614344 J Neurochem, 2003
- The  **APOE** \*4P mutation was observed in heterozygous state in four subjects, two AD 1  [ **APP** ] .71% and two controls 1.65%.  Ref: 12815750 Am J Med Genet, 2003
- Most of these studies have examined only one or two polymorphisms in a defined population, which provides a lack of a general view of the Alzheimer s disease genetic component.OBJECTIVETo overcome this limitation, nine polymorphisms in seven different candidate genes A2M, ACT,  **APOE** ,  **APP** , BH, HSP70 2, and IL1 A were genotyped.METHODSThe sample comprised 112 Alzheimer s disease patients and 89 controls from Spain.  Ref: 12782964 Psychiatr Genet, 2003
- We have examined the effect of apolipoprotein E  [ **APOE** ]  on the internalization of exogenous amyloid beta  [ **APP** ]  1 40 Abeta40 into a rat brain crude synaptosomal preparation.  Ref: 12614344 J Neurochem, 2003
- The present study was undertaken to test the hypothesis that estrogen can attenuate Ang II induced AAA  [ **APP** ]  in apolipoprotein E  [ **APOE** ]  deficient mice via its anti inflammatory mechanism.  Ref: 12855485 Arterioscler Thromb Vasc Biol< MedlineTA>Arterioscler Thromb Vasc Biol, 2003
- Ang II induced AAA  [ **APP** ]  in 9 90% of 10 hyperlipidemic mice deficient in  **apoE**   **apoE**  uPA mice but in only 2 22% of 9 mice deficient in both  **apoE**  and uPA  **apoE**  uPA mice P< ; 0.05.  Ref: 12600880 Circ Res, 2003
- We have previously demonstrated that urokinase type plasminogen activator uPA is highly expressed in the aneurysmal segment of the abdominal aorta AAA  [ **APP** ]  in apolipoprotein E  [ **APOE** ]  deficient  **apoE**  mice treated with angiotensin II Ang II.  Ref: 12600880 Circ Res, 2003
